# Side Information Design in Zero-Error Coding for Computing

**DOI:** 10.3390/e26040338

**Published:** 2024-04-16

**Authors:** Nicolas Charpenay, Maël Le Treust, Aline Roumy

**Affiliations:** 1 Univ. Rennes, CNRS, IRMAR UMR 6625, F-35000 Rennes, France; 2 Univ. Rennes, CNRS, Inria, IRISA UMR 6074, F-35000 Rennes, France; 3 INRIA Rennes, Campus de Beaulieu, F-35000 Rennes cedex, France; aline.roumy@inria.fr

**Keywords:** zero-error information theory, source coding, graph theory

## Abstract

We investigate the zero-error coding for computing problems with encoder side information. An encoder provides access to a source *X* and is furnished with side information g(Y). It communicates with a decoder that possesses side information *Y* and aims to retrieve f(X,Y) with zero probability of error, where *f* and *g* are assumed to be deterministic functions. In previous work, we determined a condition that yields an analytic expression for the optimal rate $R^{*}\left(g\right)$; in particular, it covers the case where $P_{X,Y}$ is full support. In this article, we review this result and study the side information design problem, which consists of finding the best trade-offs between the quality of the encoder’s side information g(Y) and $R^{*}\left(g\right)$. We construct two greedy algorithms that give an achievable set of points in the side information design problem, based on partition refining and coarsening. One of them runs in polynomial time.

## 1. Introduction

### 1.1. Zero-Error Coding for Computing

**Figure 1:**
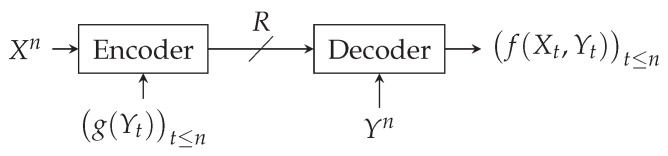
Zero-error coding for computing with side information at the encoder.

The problem of Figure 1 is a zero-error setting that relates to Orlitsky and Roche’s coding for computing problems from [1]. This coding problem appears in video compression [2,3], where $X^{n}$ models a set of images known at the encoder. The decoder does not always want to retrieve each whole image. Instead, the decoder receives, for each image $X_{t},t\leq n$, a request $Y_{t}$ to retrieve information $f(X_{t},Y_{t})$. This information can, for instance, be a detection: cat, dog, car, bike; or a scene recognition: street/city/mountain, etc. The encoder does not know the decoder’s exact request but has prior information about it (e.g., type of request), which is modeled by ${\left(g\left(Y_{t}\right)\right)}_{t\leq n}$. This problem also relates to the zero-error Slepian–Wolf open problem, which corresponds to the special case, where *g* is constant and f(X,Y)=X.

Similar schemes to the one depicted in Figure 1 have already been studied, but they differ from the one we are studying in two ways. First, they consider that no side information is available to the encoder. Second, and more importantly, they consider different coding constraints: the lossless case is studied by Orlitsky and Roche in [1], the lossy case by Yamamoto in [4], and the zero-error “unrestricted inputs” case by Shayevitz in [5]. The latter results can be used as bounds for our problem depicted in Figure 1, but do not exactly characterize its optimal rate.

Numerous extensions of the problem depicted in Figure 1 have been studied recently. The distributed context, for instance, has an additional encoder that encodes *Y* before transmitting it to the decoder. Achievability schemes have been proposed for this setting by Krithivasan and Pradhan in [6] using abelian groups; by Basu et al. in [7] using hypergraphs for the case with maximum distortion criterion; and by Malak and Médard in [8] using hyperplane separations for the continuous lossless case.

Another related context is the network setting, where the function of source random variables from source nodes has to be retrieved at the sink node of a given network. For tree networks, the feasible rate region is characterized by Feizi and Médard in [9] for networks of depth one, and by Sefidgaran and Tchamkerten in [10] under a Markov source distribution hypothesis. In [11], Ravi and Dey consider a bidirectional relay with zero-error “unrestricted inputs” and characterize the rate region for a specific class of functions. In [12], Guang et al. study zero-error function computation on acyclic networks with limited capacities, and give an inner bound based on network cut-sets. For both distributed and network settings, the zero-error coding for computing problems with encoder side information remains open.

In a previous work [13], we determined a condition that we called “pairwise shared side information” such that, if satisfied, the optimal rate $R^{*}\left(g\right)$ has a single-letter expression. This covers many cases of interest, in particular the case where $P_{X,Y}$ is full support for any functions f,g. For the sake of completeness, we review this result. Moreover, we propose an alternative and more interpretable expression for this pairwise shared side information. More precisely, we show that the instances where the “pairwise shared side information” condition is satisfied correspond to the worst possible optimal rates in an auxiliary zero-error Slepian–Wolf problem.

### 1.2. Encoder’s Side Information Design

In the zero-error coding for computing problems with encoder side information, it can be observed that a “coarse” encoder side information (e.g., if *g* constant) yields a high optimal rate $R^{*}\left(g\right)$, whereas a “fine” encoder side information (e.g., g=Id) yields a low optimal rate $R^{*}\left(g\right)$. The side information design problem consists of determining the best trade-offs between the optimal rate $R^{*}\left(g\right)$ and the quality of the encoder’s side information, which is measured by its entropy H(g(Y)). This expression describes the optimal rate of a zero-error code that transmits the quantized version of *Y* via the *g* function. The best trade-offs correspond to the Pareto front of the achievable set, i.e., whose corner-points cannot be obtained by a time sharing between other coding strategies. In short, we aim at determining the Pareto front of the convex hull of the achievable pairs $\left(H\left(g\left(Y\right)\right),R^{*}\left(g\right)\right)$.

In this article, we propose a greedy algorithm that gives an achievable set of points in the side information design problem, when $P_{X,Y}$ is full support. Studying our problem with the latter hypothesis is interesting because, unlike the case of the Slepian–Wolf problem, it does not necessarily correspond to a worst-case scenario. Recall indeed, that, when $P_{X,Y}$ is full support, the Slepian–Wolf encoder does not benefit from the side information available at the decoder and needs to send *X*. In our problem instead, if the retrieval function f(X,Y)=Y, since the decoder already has access to *Y*, no information needs to be sent by the encoder and the optimal rate is 0. Finally, the proposed algorithm relies on our results with “pairwise shared side information”, which gives the optimal rate for all functions *g* and performs a greedy partition coarsening when choosing the next achievable point. Moreover, it runs in polynomial time.

This paper is organized as follows. In Section 2, we formally present the zero-error coding for computing problems and the encoder’s side information design problem. In Section 3, we give our theoretic results on the zero-error coding for computing problems, including the “pairwise shared side information” condition. In Section 4, we present our greedy algorithms for the encoder’s side information design problem.

## 2. Formal Presentation of the Problem

We denote sequences by $x^{n}=\left(x_{1},\ldots ,x_{n}\right)$. The set of probability distributions over X is denoted by Δ(X). The distribution of *X* is denoted by $P_{X}\in \Delta \left(X\right)$ and its support is denoted by $\mathrm{supp}\;P_{X}$. Given the sequence length $n\in N^{★}$, we denote by $\Delta_{n}\left(X\right)\subset \Delta \left(X\right)$ the set of empirical distributions of sequences from $X^{n}$. We denote by ${\left\{0,1\right\}}^{*}$ the set of binary words. The collection of subsets of a set Y is denoted by P(Y).

**Definition** **1.** *The zero-error source-coding problem of Figure 1 is described by the following:*

- *Four finite sets U, X, Y, Z and a source distribution $P_{X,Y}\in \Delta \left(X\times Y\right)$.*
- *For all $n\in N^{★}$, $\left(X^{n},Y^{n}\right)$ is the random sequence of n copies of (X,Y), drawn in an i.i.d. fashion using $P_{X,Y}$.*
- *Two deterministic functions*
(1) $$\begin{array}{cc} \; & f:X\times Y\to U, \end{array}$$
(2) $$\begin{array}{cc} \; & g:Y\to Z. \end{array}$$
- *An encoder that knows $X^{n}$ and ${\left(g(Y_{t})\right)}_{t\leq n}$ sends binary strings over a noiseless channel to a decoder that knows $Y^{n}$ and that wants to retrieve ${\left(f(X_{t},Y_{t})\right)}_{t\leq n}$ without error.*

*A coding scheme in this setting is described by:*

- A time horizon $n\in N^{★}$ and an encoding function $\phi_{e}:X^{n}\times Z^{n}\to {\left\{0,1\right\}}^{*}$ such that Imϕe is prefix-free.
- A decoding function $\phi_{d}:Y^{n}\times {\left\{0,1\right\}}^{*}\to U^{n}$.
- *The rate is the average length of the codeword per source symbol,*
*i.e., $R≐\frac{1}{n}E\left[\ell \circ \phi_{e}\left(X^{n},{\left(g\left(Y_{t}\right)\right)}_{t\leq n}\right)\right]$, where ℓ denotes the codeword length function.*
- *n, $\phi_{e}$, $\phi_{d}$ must satisfy the zero-error property:*
(3) $$\begin{array}{c} P\left(\phi_{d}\left(Y^{n},\phi_{e}\left(X^{n},{\left(g\left(Y_{t}\right)\right)}_{t\leq n}\right)\right)\neq {\left(f(X_{t},Y_{t})\right)}_{t\leq n}\right)=0. \end{array}$$

*The minimal rate under the zero-error constraint is defined by*

(4) $$\begin{array}{c} R^{*}\left(g\right)≐\underset{\begin{array}{c} n,\phi_{e},\phi_{d}\\ \mathrm{zero}-\mathrm{error} \end{array}}{\inf}\frac{1}{n}E\left[\ell \circ \phi_{e}\left(X^{n},{\left(g\left(Y_{t}\right)\right)}_{t\leq n}\right)\right]. \end{array}$$

The definition of the Pareto front that we give below is adapted to the encoder’s side information design problem and allows us to describe the best trade-off between the quality of the encoder side information and the rate to compute the function f(X,Y) at the decoder. In other works, the definition of a Pareto front may differ depending on the minimization/maximization problem considered and on the number of variables to be optimized.

**Definition** **2** (Pareto front). *Let $S\subset R_{+}^{2}$ be a set, the Pareto front of S is defined by*

(5) $$\begin{array}{c} \mathrm{Par}\left(S\right)≐\left\{x\in S\;|\;\forall x^{\prime }\in S\setminus \left\{x\right\},\;x_{1}^{\prime }>x_{1}\;\mathrm{or}\;x_{2}^{\prime }>x_{2}\right\}. \end{array}$$

**Definition** **3.** The side information design problem in Figure 1 consists of determining the Pareto front of the achievable pairs $\left(H\left(g\left(Y\right)\right),R^{*}\left(g\right)\right)$:

(6) $$\begin{array}{c} F≐\mathrm{Par}\left(\mathrm{Conv}\left\{\left(H\left(g\left(Y\right)\right),R^{*}\left(g\right)\right)\;|\;g:Y\to Z\right\}\right), \end{array}$$

*where* Conv *denotes the convex hull.*

In our zero-error setup, all alphabets are finite. Therefore, the Pareto front of the convex hull in (6) is computed on a finite set of points, which correspond to the best trade-offs for the encoder’s side information.

## 3. Theoretic Results

Determining the optimal rate in the zero-error coding for computing problems, with or without encoder side information, is an open problem. In a previous contribution [13], we determined a condition that, when satisfied, yields an analytic expression for the optimal rate. Interestingly, this condition is general as it does not depend on the function *f* to be retrieved at the decoder.

### 3.1. General Case

We first build the characteristic graph $G_{\left[n\right]}$, which is a probabilistic graph that captures the zero-error encoding constraints on a given number *n* of source uses. It differs from the graphs used in [5], as we do not need a cartesian representation of these graphs to study the optimal rates. Furthermore, it has a vertex for each possible realization of $\left(X^{n},{\left(g(Y_{t})\right)}_{t\leq n}\right)$ known at the encoder, instead of $X^{n}$ as in the zero-error Slepian–Wolf problem [14].

**Definition** **4** (Characteristic graph $G_{\left[n\right]}$). *The characteristic graph $G_{\left[n\right]}$ is defined by the following:*

- *$X^{n}\times Z^{n}$ as a set of vertices with distribution $P_{X,g(Y)}^{n}$.*
- *$\left(x^{n},z^{n}\right)\left(x^{\prime n},z^{\prime n}\right)$ are adjacent if $z^{n}=z^{\prime n}$ and there exists $y^{n}\in g^{-1}\left(z^{n}\right)$ such that*
  (7) $$\begin{array}{cc} \; & \forall t\leq n,\;P_{X,Y}\left(x_{t},y_{t}\right)P_{X,Y}\left(x_{t}^{\prime },y_{t}\right)>0, \end{array}$$
  (8) $$\mathrm{and}\;\exists t\leq n,\;f\left(x_{t},y_{t}\right)\neq f\left(x_{t}^{\prime },y_{t}\right);$$
  *where $g^{-1}\left(z^{n}\right)=\left\{y^{n}\in Y^{n}\;|\;{\left(g(y_{t})\right)}_{t\leq n}=z^{n}\right\}$.*

The characteristic graph $G_{\left[n\right]}$ is designed with the same core idea as in [15]: $\left(x^{n},z^{n}\right)$ and $\left(x^{\prime n},z^{\prime n}\right)$ are adjacent if there exists a side information symbol $y^{n}$ compatible with the observation of the encoder (i.e., $z^{n}=z^{\prime n}$ and $y^{n}\in g^{-1}\left(z^{n}\right)$), such that $f\left(x^{n},y^{n}\right)\neq f\left(x^{\prime n},y^{n}\right)$. In order to prevent erroneous decodings, the encoder must map adjacent pairs of sequences to different codewords; hence the use of graph colorings, defined below.

**Definition** **5** (Coloring, independent subset). *Let $G=(V,E,P_{V})$ be a probabilistic graph. A subset S⊆V is independent if $xx^{\prime }\notin E$ for all $x,x^{\prime }\in S$. Let C be a finite set (the set of colors), a mapping c:V→C is a coloring if $c^{-1}\left(i\right)$ is an independent subset for all i∈C.*

The chromatic entropy of $G_{\left[n\right]}$ gives the best rate of *n*-shot zero-error encoding functions, as in [14].

**Definition** **6** (Chromatic entropy $H_{\chi }$). *The chromatic entropy of a probabilistic graph $G=(V,E,P_{V})$ is defined by*

(9) $$\begin{array}{c} H_{\chi }\left(G\right)=\inf\left\{H\left(c(V)\right)\;|\;c\;\mathrm{is}\;a\;\mathrm{coloring}\;\mathrm{of}\;G\right\}. \end{array}$$

**Theorem** **1** (Optimal rate). *The optimal rate is written as follows:*

(10) $$R^{*}\left(g\right)=\lim_{n\to \infty }\frac{1}{n}H_{\chi }\left(G_{\left[n\right]}\right).\;$$

**Proof.**  By construction, the following holds: for all encoding functions $\phi_{e}$, $\phi_{e}$ is a coloring of $G_{\left[n\right]}$ if and only if there exists a decoding function $\phi_{d}$ such that $\left(n,\phi_{e},\phi_{d}\right)$ satisfies the zero-error property. Thus, the best achievable rate is written as follows:

(11) $$\begin{array}{cc} R^{*}\left(g\right)= & \;\underset{n}{\inf}\underset{\phi_{e}\;\mathrm{coloring}\;\mathrm{of}\;G_{\left[n\right]}}{\inf}H\left(\phi_{e}\left(X^{n},{\left(g(Y_{t})\right)}_{t\leq n}\right)\right) \end{array}$$

(12) $$\begin{array}{cc} = & \lim_{n\to \infty }\frac{1}{n}H_{\chi }\left(G_{\left[n\right]}\right). \end{array}$$

where (12) comes from Fekete’s Lemma and from the definition of $H_{\chi }$.    □

A general single-letter expression for $R^{*}\left(g\right)$ is missing due to the lack of the intrinsic structure of $G_{\left[n\right]}$. In Section 3.2, we introduce a hypothesis that gives structure to $G_{\left[n\right]}$ and allows us to derive a single-letter expression for $R^{*}\left(g\right)$.

### 3.2. Pairwise Shared Side Information

**Definition** **7.** *The distribution $P_{X,Y}$ and the function g satisfy the “pairwise shared side information” condition if*

(13) $$\begin{array}{c} \forall z\in Z,\forall x,x^{\prime }\in X,\exists y\in g^{-1}\left(z\right),P_{XY}\left(x,y\right)P_{XY}\left(x^{\prime },y\right)>0, \end{array}$$

*where Im(g) is the image of the function g. This means that for all z output of g, every pair $\left(x,x^{\prime }\right)$ “shares” at least one side information symbol $y\in g^{-1}\left(z\right)$.*

Note that any full-support distribution $P_{X,Y}$ satisfies the “pairwise shared side information” hypothesis. In Theorem 2, we give an interpretation of the “pairwise shared side information” condition in terms of the optimal rate in an auxiliary zero-error Slepian–Wolf problem.

**Theorem** **2.** *The tuple $\left(P_{X,Y},g\right)$ satisfies the condition “pairwise shared side information” (13)*

* ⟺* $R^{*}\left(g\right)=H\left(X|g\left(Y\right)\right)$ *in the case* f(X,Y)=X*, and for all* z∈Z*,* $P_{X|g(Y)=z}$ *is full support.*

The proof of Theorem 2 is given in Appendix A.1.

**Definition** **8** (AND, OR product). *Let $G_{1}\;=\;\left(V_{1},E_{1},P_{V_{1}}\right)$, $G_{2}=\left(V_{2},E_{2},P_{V_{2}}\right)$ be two probabilistic graphs; their AND (resp. OR) product denoted by $G_{1}\wedge G_{2}$ (resp. $G_{1}\vee G_{2}$) is defined by the following: $V_{1}\times V_{2}$ as a set of vertices, $P_{V_{1}}P_{V_{2}}$ as probability distribution on the vertices, and $\left(v_{1}v_{2}\right),\left(v_{1}^{\prime }v_{2}^{\prime }\right)$ are adjacent if*

(14) $$\begin{array}{c} v_{1}v_{1}^{\prime }\in E_{1}\;\mathrm{AND}\;v_{2}v_{2}^{\prime }\in E_{2},\\ \mathrm{resp}.\;(v_{1}v_{1}^{\prime }\in E_{1}\;\mathrm{and}\;v_{1}\neq v_{1}^{\prime })\;\mathrm{OR}\;(v_{2}v_{2}^{\prime }\in E_{2}\;\mathrm{and}\;v_{2}\neq v_{2}^{\prime }); \end{array}$$

*with the convention that all vertices are self-adjacent. We denote by $G_{1}^{\wedge n}$ (resp. $G_{1}^{\vee n}$) the n-th AND (resp. OR) power.*

AND and OR powers significantly differ in terms of existing single-letter expression for the associated asymptotic chromatic entropy. Indeed, in the zero-error Slepian–Wolf problem in [14], the optimal rate $\lim_{n\to \infty }\frac{1}{n}H_{\chi }\left(G^{\wedge n}\right)$, which relies on an AND power, does not have a single-letter expression. Instead, closed-form expressions for OR powers of graphs exist. More precisely, as recalled in Proposition 1, $\lim_{n\to \infty }\frac{1}{n}H_{\chi }\left(G^{\vee n}\right)$ admits a single-letter expression called the Körner graph entropy, introduced in [16], and defined below. This observation is key for us to derive a single-letter expression for our problem. More precisely, by using a convex combination of Körner graph entropies, we provide a single-letter expression in Theorem 3 for the optimal rate $R^{*}\left(g\right)$.

**Definition** **9** (Körner graph entropy $H_{\kappa }$). *For all $G=(V,E,P_{V})$, let Γ(G) be the collection of independent sets of vertices in G. The Körner graph entropy of G is defined by*

(15) $$\begin{array}{c} H_{\kappa }\left(G\right)=\min_{V\in W\in \Gamma (G)}I\left(W;V\right), \end{array}$$

*where the minimum is taken over all distributions $P_{W|V}\in \Delta {\left(W\right)}^{V}$, with W=Γ(G) and the constraint that the random vertex V belongs to the random set W with probability one.*

Below, we recall that the limit of the normalized chromatic entropy of the OR product of graphs admits a closed-form expression given by the Körner graph entropy $H_{\kappa }$. Moreover, the Körner graph entropy of OR products of graphs is simply the sum of the individual Körner graph entropies.

**Proposition** **1** (Properties of $H_{\kappa }$). *Theorem 5 in [14] for all probabilistic graphs G and $G^{\prime }$,*

(16) $$\begin{array}{cc} \; & H_{\kappa }\left(G\right)=\lim_{n\to \infty }\frac{1}{n}H_{\chi }\left(G^{\vee n}\right), \end{array}$$

(17) $$\begin{array}{cc} \; & H_{\kappa }\left(G\vee G^{\prime }\right)=H_{\kappa }\left(G\right)+H_{\kappa }\left(G^{\prime }\right). \end{array}$$

**Definition** **10** (Auxiliary graph $G_{z}^{f}$). *For all z∈Z, we define the auxiliary graph $G_{z}^{f}$ by*

- *X as set of vertices with distribution $P_{X|g(Y)=z}$;*
- *$xx^{\prime }$ are adjacent if $f\left(x,y\right)\neq f\left(x^{\prime },y\right)$ for some $y\in g^{-1}\left(z\right)\cap \mathrm{supp}P_{Y|X=x}\cap \mathrm{supp}P_{Y|X=x^{\prime }}$.*

**Theorem** **3** (Pairwise shared side information). *If $P_{X,Y}$ and g satisfy (13), the optimal rate is written as follows:*

(18) $$R^{*}\left(g\right)=\sum_{z\in Z}P_{g(Y)}\left(z\right)H_{\kappa }\left(G_{z}^{f}\right).$$

The proof is in Appendix A.2, the keypoint is the particular structure of $G_{\left[n\right]}$: a disjointed union of OR products.

**Remark** **1.** The “pairwise shared side information” assumption (13) implies that the adjacency condition (7) is satisfied, which makes $G_{\left[n\right]}$ a disjoint union of OR products. Moreover, Körner graph entropies appear in the final expression for $R^{*}\left(g\right)$, even if $G_{\left[n\right]}$ is not an n-th OR power.

Now, consider the case where $P_{X,Y}$ is full support. This is a sufficient condition to have (13). The optimal rate in this setting is derived from Theorem 3, which leads to the analytic expression in Theorem 4.

**Theorem** **4** (Optimal rate when $P_{X,Y}$ is full support). *When $P_{X,Y}$ is full support, the optimal rate is written as follows:*

(19) $$\begin{array}{cc} R^{*}\left(g\right)= & \;H\left(j(X,g(Y))|g(Y)\right), \end{array}$$

where the function j returns a word in $U^{*}$, defined by

(20) $$\begin{array}{cc} j: & \;X\times Z\to U^{*}\\ \; & \left(x,z\right)\mapsto {\left(f(x,y^{\prime })\right)}_{y^{\prime }\in g^{-1}\left(z\right)}. \end{array}$$

**Proof.**  By Theorem 3, $R^{*}\left(g\right)=\sum_{z\in Z}P_{g(Y)}\left(z\right)H_{\kappa }\left(G_{z}^{f}\right)$. It can be shown that $G_{z}^{f}$ is complete multipartite for all *z* as $P_{X,Y}$ is full support; and it satisfies $H_{\kappa }\left(G_{z}^{f}\right)=H\left(j(X,g(Y))|g(Y)=z\right)$.    □

### 3.3. Example

In this example, the “pairwise shared side information” assumption is satisfied and $R^{*}\left(g\right)$ is strictly less than a conditional Huffman coding of *X* knowing g(Y); and also strictly less than the optimal rate without exploiting g(Y) at the encoder.

**Figure 2:**
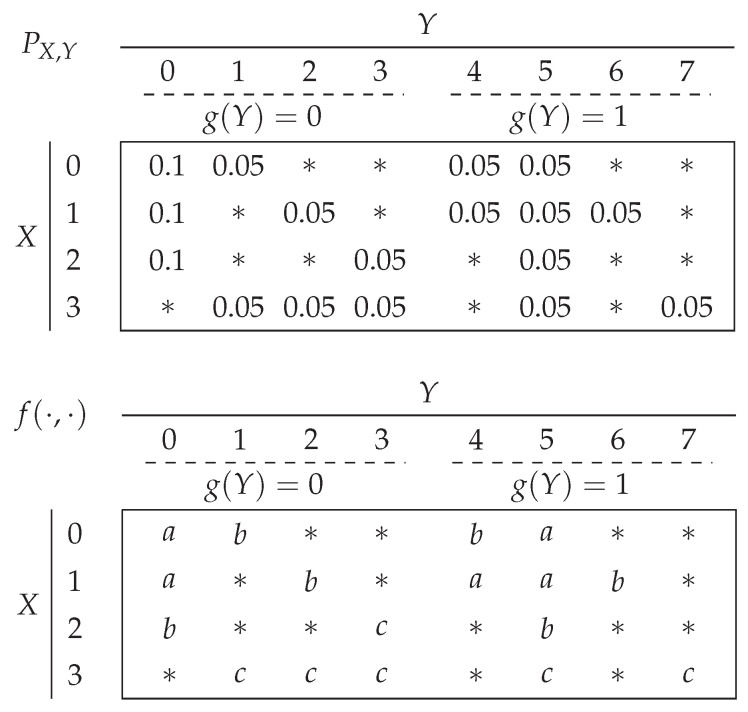
An example of $P_{X,Y}$ and *g* that satisfies (13), along with the outcomes f(X,Y). The elements outside $\mathrm{supp}P_{X,Y}$ are denoted by *.

Consider the probability distribution and function outcomes depicted in Figure 2, with U={a,b,c}, X={0,…,3}, Y={0,…,7}, and Z={0,1}. Let us show that the “pairwise shared side information” assumption is satisfied. The source symbols 0,1,2∈X share the side information symbol 0 (resp. 5) when g(Y)=0 (resp. g(Y)=1). The source symbol 3∈X shares the side information symbols 1,2,3 with the source symbols 0,1,2, respectively, when g(Y)=0, and the source symbol 3 shares the side information symbol 5 with all other source symbols when g(Y)=1.

Since the “pairwise shared side information” assumption is satisfied, we can use Theorem 3; the optimal rate is written as follows:

(21) $$\begin{array}{c} R^{*}\left(g\right)=P_{g(Y)}\left(0\right)H_{\kappa }\left(G_{0}^{f}\right)+P_{g(Y)}\left(1\right)H_{\kappa }\left(G_{1}^{f}\right). \end{array}$$

First, we need to determine the probabilistic graphs $G_{0}^{f}$ and $G_{1}^{f}$. In $G_{0}^{f}$, the vertex 0 is adjacent to 2 and 3, as f(0,0)≠f(2,0) and f(0,1)≠f(3,1). The vertex 1 is also adjacent to 2 and 3 as f(1,0)≠f(2,0) and f(1,2)≠f(3,2). Furthermore $P_{X|g(Y)=0}$ is uniform, hence $G_{0}^{f}=\left(C_{4},\mathrm{Unif}\left(X\right)\right)$ where $C_{4}$ is the cycle graph with 4 vertices.

In $G_{1}^{f}$, the vertices 1, 2, 3 are pairwise adjacent as f(1,5), f(2,5) and f(3,5) are pairwise different; and 0 is adjacent to 1, 2, and 3 because of the different function outputs generated by Y=4 and Y=5. Thus, $G_{1}^{f}=\left(K_{4},P_{X|g(Y)=1}\right)$ with $P_{X|g(Y)=1}=\left(\frac{1}{4},\frac{3}{8},\frac{1}{8},\frac{1}{4}\right)$, and $K_{4}$ is the complete graph with 4 vertices.

Now, let us determine $H_{\kappa }\left(G_{0}^{f}\right)$ and $H_{\kappa }\left(G_{1}^{f}\right)$. On the one hand,

(22) $$\begin{array}{cc} H_{\kappa }\left(G_{0}^{f}\right) & =H\left(V_{0}\right)-\max_{V_{0}\in W\in \Gamma \left(G_{0}^{f}\right)}H\left(V_{0}|W\right) \end{array}$$

(23) $$\begin{array}{cc} \; & =2-1=1, \end{array}$$

with $V_{0}∼P_{X|g(Y)=0}=\mathrm{Unif}\left(X\right)$; and where $H(V_{0}|W)$ in (22) is maximized by taking W={0,1} when V∈{0,1}, and W={2,3} otherwise.

On the other hand,

(24) $$\begin{array}{cc} H_{\kappa }\left(G_{1}^{f}\right) & =\min_{V_{1}\in W\in \Gamma \left(G_{1}^{f}\right)}I\left(W;V_{1}\right) \end{array}$$

(25) $$\begin{array}{cc} \; & =H(V_{1})\approx 1.906, \end{array}$$

with $V_{1}∼P_{X|g(Y)=1}$; where (25) follows from $\Gamma \left(G_{1}^{f}\right)=\left\{\left\{0\right\},\ldots ,\left\{3\right\}\right\}$, as $G_{1}^{f}$ is complete. Hence, $R^{*}\left(g\right)\approx 1.362$.

The rate that we would obtain by transmitting *X* knowing g(Y) at both encoder and decoder with a conditional Huffman algorithm is written as $R_{\mathrm{Huff}}=H\left(X|g\left(Y\right)\right)\approx 1.962$.

The rate that we would obtain without exploiting g(Y) at the encoder is $R_{\mathrm{No}\;g}=H\left(X\right)\approx 1.985$ because of the different function outputs generated by Y=4 and Y=5.

Finally, H(f(X,Y)|Y)≈0.875.

In this example, we have

(26) $$\begin{array}{c} H\left(X\right)=R_{\mathrm{No}\;g}>R_{\mathrm{Huff}}>R^{*}\left(g\right)>H\left(f\left(X,Y\right)|Y\right). \end{array}$$

This illustrates the impact of the side information at the encoder in this setting, as we can observe a large gap between the optimal rate $R^{*}\left(g\right)$ and $R_{\mathrm{No}\;g}$.

## 4. Optimization of the Encoder Side Information

### 4.1. Preliminary Results on Partitions

In order to optimize the function *g* in the encoder side information, we propose a new equivalent characterization of the function *g* in the form of a partition of the set Y. The equivalence is shown in Proposition 2 below.

**Proposition** **2.** *For all g:Y→Z, the collection of subsets ${\left(g^{-1}\left(z\right)\right)}_{z\in Z}$ is a partition of Y.*

*Conversely, if A⊂P(Y) is a partition of Y, then there exists a mapping $g_{A}:Y\to Z$ such that ∀z∈ImgA,∃Az∈A,Az=gA−1(z).*

**Proof.**  The direct part results directly from the fact that *g* is a function. For the converse part, we take Z such that |Z|=|A| and we define $g_{A}:Y\to Z$ by $g_{A}\left(y\right)=z$, where z∈Z is the unique index such that $y\in A_{z}$. The property ∀z∈ImgA,∃Az∈A,Az=gA−1(z) is therefore satisfied.    □

Now, let us define coarser and finer partitions, with the corresponding notions of merging and splitting. These operations on partitions are the core idea of our greedy algorithms; as illustrated in Proposition 2, the partitions of Y correspond to functions g:Y→Z for the encoder’s side information. Therefore, obtaining a partition from another means finding another function g:Y→Z for the encoder’s side information.

**Definition** **11** (Coarser, Finer). *Let A,B⊂P(Y) be two partitions of the finite set Y. We say that A is coarser than B if*

(27) $$\begin{array}{c} \forall B\in B,\;\exists A\in A,\;B\subset A. \end{array}$$

*If so, we also say that B is finer than A.*

**Example** **1.** *Let Y={1,2,3,4}, the partition $A=\left\{\{1\},\{2,3,4\}\right\}$ is coarser than $B=\left\{\{1\},\{2\},\{3,4\}\right\}$.*

**Definition** **12** (Merging, Splitting). *A merging is an operation that maps a partition $A=\{A_{1},\ldots ,A_{i},\ldots ,A_{j},\ldots ,A_{m}\}$ to the partition $A^{\prime }=\left\{A_{1},\ldots ,A_{i}\cup A_{j},\ldots ,A_{m}\right\}$. A splitting in an operation that maps a partition $A=\{A_{1},\ldots ,A_{i},\ldots ,A_{m}\}$ to the partition $A^{\prime }=\left\{A_{1},\ldots ,A_{i}^{\left(1\right)},A_{i}^{\left(2\right)},\ldots ,A_{m}\right\}$, where $\left\{A_{i}^{\left(1\right)},A_{i}^{\left(2\right)}\right\}$ form a partition of the subset $A_{i}$.*

*We also define the set of partitions Merge(A) (resp. Split(A)), which correspond to all partitions that can be obtained with a merging (resp. splitting) of A:*

(28) $$\begin{array}{cc} \; & \mathrm{Merge}\left(A\right)≐\left\{m(A)\;|\;m\;\mathrm{is}\;a\;\mathrm{merging}\right\}; \end{array}$$

(29) $$\begin{array}{cc} \; & \mathrm{Split}\left(A\right)≐\left\{s(A)\;|\;s\;\mathrm{is}\;a\;\mathrm{splitting}\right\}. \end{array}$$

**Proposition** **3.** *If A is coarser (resp. finer) than B, then A can be obtained from B by performing a finite number of mergings (resp. splittings).*

### 4.2. Greedy Algorithms Based on Partition Coarsening and Refining

In this Section, we assume $P_{X,Y}$ to be full support.

With Proposition 2, we know that determining the Pareto front by a brute force approach would at least require to enumerate the partitions of Y. Therefore, the complexity of this approach is exponential in |Y|. In the following we describe the greedy Algorithms 1 and 2 that give an achievable set for the encoder’s side information design problem; one of them has a polynomial complexity. Then we give an example where the Pareto front coincides with the boundary of the convex hull of the achievable rate region obtained by both greedy algorithms.
**Algorithm 1** Greedy coarsening algorithm1:$A\leftarrow \left\{\{1\},\ldots \{|Y|\}\right\}$       // A starts by being the finest partition of Y, i.e., $g_{A}=\mathrm{Id}$.2:*Front*←[A,ndef,…,ndef]       // Will contain the list of the |Y| partitions chosen during the execution3: 4:**for** i∈{1,…,|Y|−1} **do**5:    // Maximize over B merging of A the slope between $\left(H\left(g_{B}\left(Y\right)\right),R^{*}\left(g_{B}\right)\right)$ and $\left(H\left(g_{A}\left(Y\right)\right),R^{*}\left(g_{A}\right)\right)$.6:    $A\leftarrow {\mathrm{argmax}}_{B\in \mathrm{Merge}(A)}\frac{R^{*}\left(g_{B}\right)-R^{*}\left(g_{A}\right)}{H\left(g_{B}\left(Y\right)\right)-H\left(g_{A}\left(Y\right)\right)}$7:    *Front*[i]←A8: 9:**return** *Front*       // $A=\left\{\{1,\ldots ,|Y|\}\right\}$ at this point

**Algorithm 2** Greedy refining algorithm
1:$A\leftarrow \left\{\{1,\ldots ,|Y|\}\right\}$       // A starts by being the coarsest partition of Y, i.e., $g_{A}=\mathrm{Id}$.2:*Front* ←[A,ndef,…,ndef]       // Will contain the list of the |Y| partitions chosen during the execution3: 4:**for** i∈{1,…,|Y|−1} **do**5:    // Minimize over B splitting of A the slope between $\left(H\left(g_{A}\left(Y\right)\right),R^{*}\left(g_{A}\right)\right)$ and $\left(H\left(g_{B}\left(Y\right)\right),R^{*}\left(g_{B}\right)\right)$.6:    $A\leftarrow {\mathrm{argmin}}_{B\in \mathrm{Split}(A)}\frac{R^{*}\left(g_{B}\right)-R^{*}\left(g_{A}\right)}{H\left(g_{B}\left(Y\right)\right)-H\left(g_{A}\left(Y\right)\right)}$7:    *Front*[i]←A8: 9:**return** *Front*      // $A=\left\{\{1\},\ldots \{|Y|\}\right\}$ at this point


In these a argmin (resp. argmax) means any minimizer (resp. maximizer) of the specified quantity; and the function $g_{A}:Y\to Z$ is a function for the encoder’s side information corresponding to the partition A, whose existence is given by Proposition 2.

The coarsening (resp. refining) algorithm starts by computing its first achievable point $\left(H\left(g_{A}\left(Y\right)\right),R^{*}\left(g_{A}\right)\right)$ with A being the finest (resp. coarsest) partition: it evaluates $R^{*}\left(g_{A}\right)$, with $g_{A}=\mathrm{Id}$ (resp. $g_{A}$ constant); and $H(g_{A}\left(Y\right))=H\left(Y\right)$ (resp. $H(g_{A}\left(Y\right))=0$). Then, at each iteration, the next achievable point will be computed by using a merging (resp. splitting) of the current partition A. The next partition will be a coarser (resp. finer) partition chosen from Merge(A) (resp. Split(A)), following a greedy approach. Since we want to achieve good trade-offs between $H(g_{A}\left(Y\right))$ and $R^{*}\left(g_{A}\right)$, we want to decrease H(g(Y)) (resp. $R^{*}\left(g_{A}\right)$) as much as possible while increasing the other quantity as less as possible. We do so by maximizing over B∈Merge(A) the negative ratio

(30) $$\begin{array}{c} \frac{R^{*}\left(g_{B}\right)-R^{*}\left(g_{A}\right)}{H\left(g_{B}\left(Y\right)\right)-H\left(g_{A}\left(Y\right)\right)}, \end{array}$$

resp. minimizing over B∈Split(A) the negative ratio

(31) $$\begin{array}{c} \frac{R^{*}\left(g_{B}\right)-R^{*}\left(g_{A}\right)}{H\left(g_{B}\left(Y\right)\right)-H\left(g_{A}\left(Y\right)\right)}; \end{array}$$

hence the use of slope maximization (resp. minimization) in the algorithm. At the end, the set of achievable points computed by the algorithm is returned.

**Figure 3:**
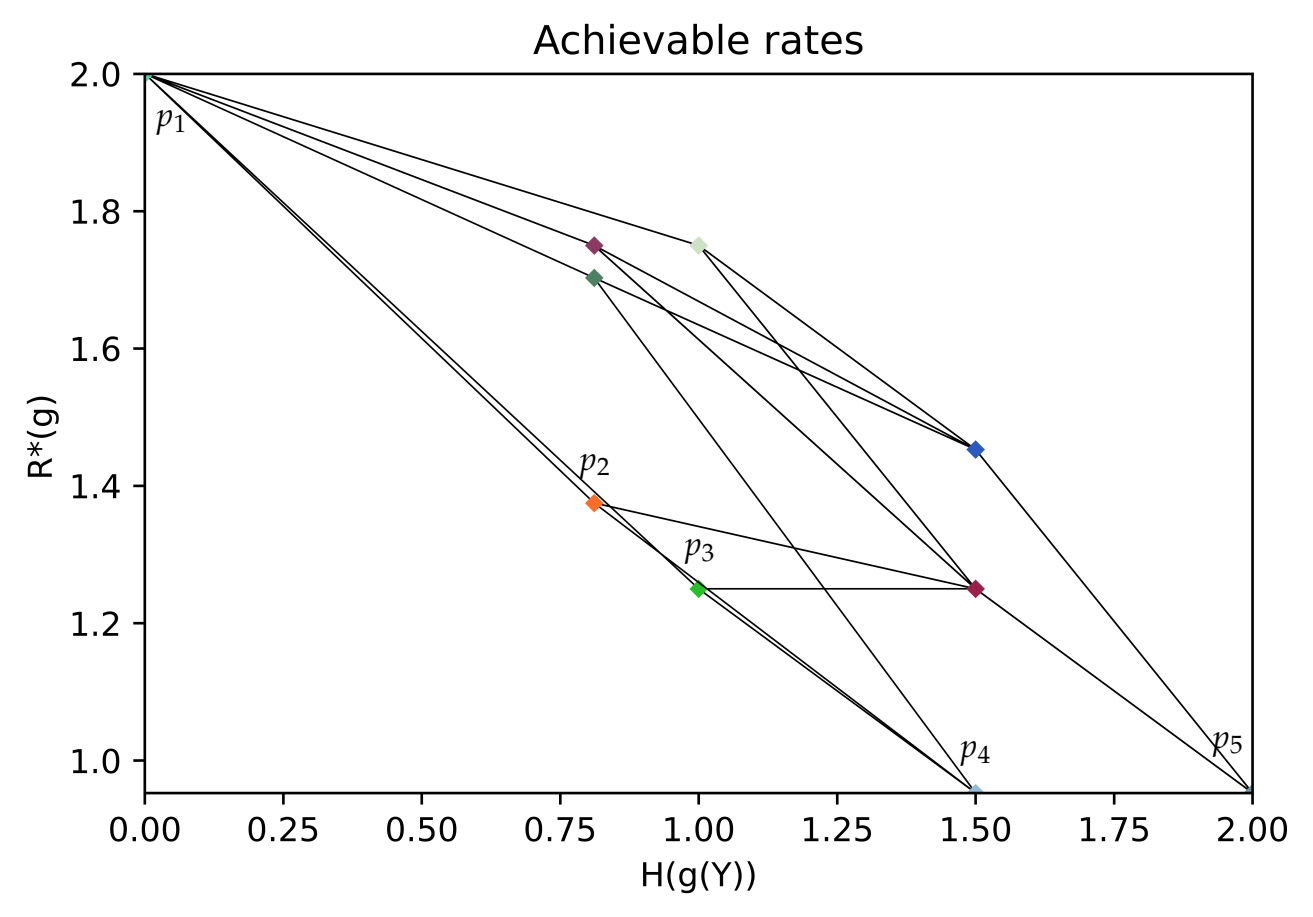
An illustration of the rate pairs associated with all partitions of Y. The Pareto front is the broken line corresponding to the partitions $p_{1}$–$p_{2}$–$p_{3}$–$p_{4}$–$p_{5}$; with $p_{1}=\left\{\{1,2,3,4\}\right\}$, $p_{2}=\left\{\{1,2,4\},\{3\}\right\}$, $p_{3}=\left\{\{1,2\},\{3,4\}\right\}$, $p_{4}=\left\{\{1,2\},\{3\},\{4\}\right\}$, $p_{5}=\left\{\{1\},\{2\},\{3\},\{4\}\right\}$.

**Figure 4:**
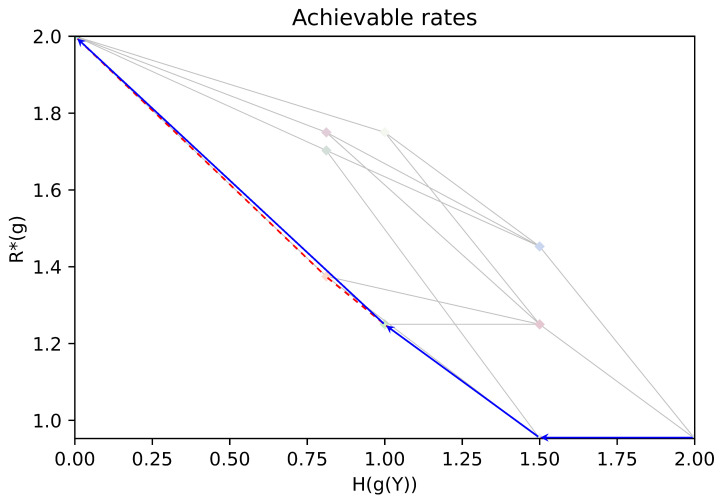
An illustration of the trajectory of the coarsening greedy algorithm (blue), with the Pareto front of the achievable rates (dashed red).

**Figure 5:**
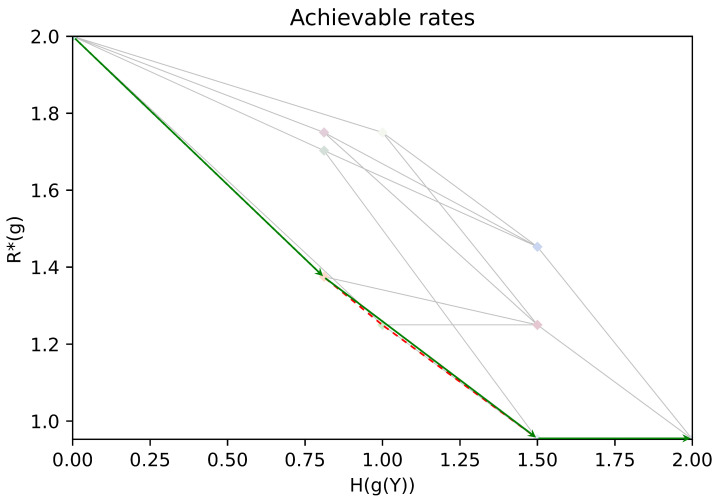
An illustration of the trajectory of the refining greedy algorithm (green), with the Pareto front of the achievable rates (dashed red).

In Figure 3, we show rate pairs associated with all possible partitions of Y: a point corresponds to a partition of Y, its position gives the associated rates $\left(H\left(g\left(Y\right)\right),R^{*}\left(g\right)\right)$. Two points are linked if their corresponding partitions A,B satisfy A∈Merge(B) or A∈Split(B). The obtained graph is the Hasse diagram for the partial order “coarser than”. Note that due to symmetries in the chosen example, several points associated with different partitions may overlap. In Figure 4, (resp. Figure 5), we give an illustration of the trajectory of the greedy coarsening (resp. refining) algorithm.

Figure 3, Figure 4 and Figure 5 are obtained with the following problem data:

(32) $$\begin{array}{c} P_{X,Y}=\mathrm{Unif}\left(X\times Y\right)\;f\left(\cdot ,\cdot \right)=\left(\begin{array}{cccc} 0 & 0 & 0 & 1\\ 0 & 0 & 1 & 1\\ 1 & 1 & 0 & 0\\ 1 & 1 & 1 & 1 \end{array}\right). \end{array}$$

As stated in Theorem 5, the complexity of the coarsening greedy algorithm is polynomial since |Merge(A)| is quadratic in |Y| and the evaluation of $R^{*}\left(g\right)$ can be conducted in polynomial time. This polynomial complexity property is not satisfied by the refining greedy algorithm, as |Split(A)| is exponential in |Y|.

**Theorem** **5.** *The coarsening greedy algorithm runs in polynomial time in |Y|. The refining greedy algorithm runs in exponential time in |Y|.*

**Proof.**  The number of points evaluated by the coarsening (resp. refining) greedy algorithm is $O(|Y{|}^{3})$ (resp. $O(2^{\left|Y\right|})$): O(|Y|) mergings (resp. splittings) are made; and for each of these mergings, all points from Merge(A) (resp. Split(A)) are evaluated; they are, at most, (|Y|2)=O(|Y|2) (resp. $O(2^{\left|Y\right|})$, in the worst case $A=\left\{\{1,\ldots ,|Y|\}\right\}$). Since the expression $R^{*}\left(g\right)=H\left(j(X,g(Y))|g(Y)\right)$ from Theorem 4 allows for an evaluation of $R^{*}\left(g\right)$ in polynomial time in |Y|, the coarsening (resp. refining) greedy algorithm has a polynomial (resp. exponential) time complexity.    □

## Figures and Tables

**Figure A1:**
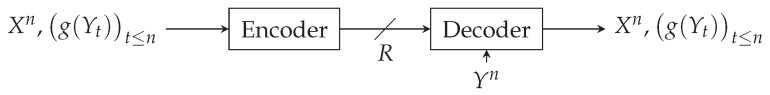
An auxiliary zero-error Slepian–Wolf problem.

## Data Availability

No new data were created or analyzed in this study. Data sharing is not applicable to this article.

## Appendix A

### Appendix A.1. Proof of Theorem 2

Consider the particular case f(X,Y)=X of Figure 1. The optimal rate in this particular case equals the optimal rate $R^{*}\left(g\right)$ in the following auxiliary problem, depicted in Figure A1: (X,g(Y)) as source available at the encoder and to be retrieved by the decoder which knows *Y* (thus, expecting it to retrieve g(Y) in addition to *X* does not change the optimal rate).

**Definition** **A1** (Characteristic graph for the zero-error Slepian–Wolf problem). *Let X,Y be two finite sets and $P_{Y|X}$ be a conditional distribution from $\Delta {\left(Y\right)}^{\left|X\right|}$. The characteristic graph associated with $P_{Y|X}$ is defined by the following:*

- *X as set of vertices;*
- *$x,x^{\prime }\in X$ are adjacent if $P_{Y|X}\left(y|x\right)P_{Y|X}\left(y|x^{\prime }\right)>0$ for some y∈Y.*

This auxiliary problem is a particular instance of the zero-error Slepian–Wolf problem; its optimal rate is written as $\bar{H}\left(G\right)$, where $\bar{H}\left(G\right)$ is the complementary graph entropy [17] and *G* is the characteristic graph in the Slepian–Wolf problem, defined in Definition A1, for the pair $\left((X,g(Y)),Y\right)$. The graph *G* has X×Z as a set of vertices, and (x,z) is adjacent to $\left(x^{\prime },z^{\prime }\right)$ if there exists a side information symbol y∈Y such that $P_{X,Y,g(Y)}\left(x,y,z\right)P_{X,Y,g(Y)}\left(x^{\prime },y,z^{\prime }\right)>0$. It can be observed that the vertices (x,z) and $\left(x^{\prime },z^{\prime }\right)$ such that $z\neq z^{\prime }$ are not adjacent in *G*. The graph *G* is therefore a disjoint union indexed by Z:

(A1) $$\begin{array}{cc} \; & G={⨆}_{z\in Z}^{P_{g(Y)}}G_{z}; \end{array}$$

(A2) $$\begin{array}{cc} \; & R^{*}\left(g\right)=\bar{H}\left(G\right)=\bar{H}\left({⨆}_{z\in Z}^{P_{g(Y)}}G_{z}\right)\;; \end{array}$$

where for all z∈Z, $G_{z}$ is the characteristic graph defined in Definition A1 for the pair $\left(X_{z}^{\prime },Y_{z}^{\prime }\right)∼P_{X,Y|g(Y)=z}$.

(⇒) Assume that *g* and $P_{X,Y}$ satisfy the “pairwise shared side information” condition. It directly follows that $P_{X|g(Y)=z}$ is full support for all z∈Z. Let z∈Z, and let $\left(x,z\right),\left(x^{\prime },z\right)$ be any two vertices of $G_{z}$. By construction, there exists $y\in g^{-1}\left(z\right)$ such that $P_{X,Y}\left(x,y\right)P_{X,Y}\left(x^{\prime },y\right)>0$; hence, $P_{X,Y,g(Y)}\left(x,y,z\right)P_{X,Y,g(Y)}\left(x^{\prime },y,z\right)>0$, and $\left(x,z\right),\left(x^{\prime },z\right)$ are adjacent in $G_{z}$. Each graph $G_{z}$ is therefore complete and perfect; the graph $G={⨆}_{z\in Z}^{P_{g(Y)}}G_{z}$ is a disjoint union of perfect graphs and is therefore also perfect. We have the following:

(A3) $$\begin{array}{cc} R^{*}\left(g\right) & =\bar{H}\left({⨆}_{z\in Z}^{P_{g(Y)}}G_{z}\right) \end{array}$$

(A4) $$\begin{array}{cc} \; & =H_{\kappa }\left({⨆}_{z\in Z}^{P_{g(Y)}}G_{z}\right) \end{array}$$

(A5) $$\begin{array}{cc} \; & =\sum_{z\in Z}P_{g(Y)}\left(z\right)H_{\kappa }\left(G_{z}\right) \end{array}$$

(A6) $$\begin{array}{cc} \; & =\sum_{z\in Z}P_{g(Y)}\left(z\right)H\left(P_{X|g(Y)=z}\right) \end{array}$$

(A7) $$\begin{array}{cc} \; & =H(X|g(Y)); \end{array}$$

where (A3) comes from (A2); (A4) and (A5) follow from Corollary 12 in [18] used on the perfect graph ${⨆}_{z\in Z}^{P_{g(Y)}}G_{z}$; and (A6) holds as the independent subsets of the complete graph $G_{z}$ are singletons containing one of its vertices.

(⇐) Conversely, assume that $P_{X|g(Y)=z}$ is full support for all z∈Z, and $R^{*}\left(g\right)=H\left(X|g\left(Y\right)\right)$.

Assume, ad absurdum, that at least one of the ${\left(G_{z}\right)}_{z\in Z}$ is not complete; then, there exists a coloring of that graph that maps two different vertices to the same color. Thus, there exists z∈Z such that

(A8) $$\begin{array}{c} \bar{H}\left(G_{z}\right)<H\left(P_{X|g(Y)=z}\right), \end{array}$$

as $P_{X|g(Y)=z}$ is full support. We have

(A9) $$\begin{array}{cc} H(X|g(Y)) & =R^{*}\left(g\right) \end{array}$$

(A10) $$\begin{array}{cc} \; & =\bar{H}\left({⨆}_{z\in Z}^{P_{g(Y)}}G_{z}\right) \end{array}$$

(A11) $$\begin{array}{cc} \; & \leq \sum_{z\in Z}P_{g(Y)}\left(z\right)\bar{H}\left(G_{z}\right) \end{array}$$

(A12) $$\begin{array}{cc} \; & <H(X|g(Y)); \end{array}$$

where (A10) comes from (A2), (A11) results from Theorem 2 in [17], and (A12) follows from (A8). We arrive at a contradiction, and hence all the graphs ${\left(G_{z}\right)}_{z\in Z}$ are complete: for all z∈Z and $x,x^{\prime }\in X$, there exists a side information symbol y∈Y such that $P_{X,Y,g(Y)}\left(x,y,z\right)P_{X,Y,g(Y)}\left(x^{\prime },y,z\right)>0$; hence, $y\in g^{-1}\left(z\right)$ and satisfies $P_{X,Y}\left(x,y\right)P_{X,Y}\left(x^{\prime },y\right)>0$. The condition “pairwise shared side information” is satisfied by $P_{X,Y},g$.

### Appendix A.2. Proof of Theorem 3

Let us specify the adjacency condition in $G_{\left[n\right]}$ under assumption (13). Two vertices are adjacent if they satisfy (7) and (8); however, (7) is always satisfied under (13). Thus, $\left(x^{n},z^{n}\right)\left(x^{\prime n},z^{n}\right)$ are adjacent if $z^{n}=z^{\prime n}$ and

(A13) $$\begin{array}{c} \exists y^{n}\in g^{-1}\left(z^{n}\right),\exists t\leq n,f\left(x_{t},y_{t}\right)\neq f\left(x_{t}^{\prime },y_{t}\right). \end{array}$$

It can be observed that condition (A13) is the adjacency condition of an OR product of adequate graphs; more precisely,

(A14) $$\begin{array}{c} G_{\left[n\right]}=\underset{z^{n}\in Z^{n}}{⨆}\underset{t\leq n}{⋁}G_{z_{t}}^{f}. \end{array}$$

Although $G_{\left[n\right]}$ cannot be expressed as an *n*-th OR power, we will show that its chromatic entropy asymptotically coincides with that of an appropriate OR power: we now search for an asymptotic equivalent of $H_{\chi }\left(G_{\left[n\right]}\right)$.

**Definition** **A2.**
*$S_{n}$ is the set of colorings of $G_{\left[n\right]}$ that can be written as $\left(x^{n},z^{n}\right)\mapsto \left(T_{z^{n}},\tilde{c}\left(x^{n},z^{n}\right)\right)$ for some mapping $\tilde{c}:X^{n}\times Z^{n}\to \tilde{C}$; where $T_{z^{n}}$ denotes the type of $z^{n}$.*

In the following, we define $Z^{n}≐{\left(g(Y_{t})\right)}_{t\leq n}$. Now, we need several Lemmas. Lemma A1 states that the optimal coloring $c(x^{n},z^{n})$ of $G_{\left[n\right]}$ has the type of $z^{n}$ as a prefix at a negligible rate cost. Lemma A3 gives an asymptotic formula for the minimal entropy of the colorings from $S_{n}$.

**Lemma** **A1.**
*The following asymptotic comparison holds as follows:*

(A15)
$$\begin{array}{c} H_{\chi }\left(G_{\left[n\right]}\right)=\underset{\begin{array}{c} c\;\mathrm{coloring}\;\mathrm{of}\;G_{\left[n\right]}\\ s.t.\;c\in S_{n} \end{array}}{\inf}H\left(c\left(X^{n},Z^{n}\right)\right)+O\left(\log n\right). \end{array}$$

**Definition** **A3** (Isomorphic probabilistic graphs). *Let $G_{1}=\left(V_{1},E_{1},P_{V_{1}}\right)$ and $G_{2}=\left(V_{2},E_{2},P_{V_{2}}\right)$ be two probabilistic graphs. We say that $G_{1}$ is isomorphic to $G_{2}$ (denoted by $G_{1}≃G_{2}$) if there exists an isomorphism between them, i.e., a bijection $\psi :V_{1}\to V_{2}$ such that*

- *For all $v_{1},v_{1}^{\prime }\in V_{1}$, $v_{1}v_{1}^{\prime }\in E_{1}⟺\psi \left(v_{1}\right)\psi \left(v_{1}^{\prime }\right)\in E_{2}$;*
- *For all $v_{1}\in V_{1}$, $P_{V_{1}}\left(v_{1}\right)=P_{V_{2}}\left(\psi (v_{1})\right)$.*

**Lemma** **A2.**
*Let B be a finite set, let $P_{B}\in \Delta \left(B\right)$ and let ${\left(G_{b}\right)}_{b\in B}$ be a family of isomorphic probabilistic graphs, then $H_{\chi }\left({⨆}_{b^{\prime }\in B}^{P_{B}}G_{b^{\prime }}\right)=H_{\chi }\left(G_{b}\right)$ for all b∈B.*

**Lemma** **A3.**
*The following asymptotic comparison holds as follows:*

(A16)
$$\begin{array}{c} \underset{\begin{array}{c} c\;\mathrm{coloring}\;\mathrm{of}\;G_{\left[n\right]}\\ s.t.\;c\in S_{n} \end{array}}{\inf}H\left(c\left(X^{n},Z^{n}\right)\right)=n\sum_{z\in Z}P_{g(Y)}\left(z\right)H_{\kappa }\left(G_{z}^{f}\right)+o\left(n\right). \end{array}$$

The proof of Lemma A1 is given in Appendix A.3 and its keypoint is the asymptotically negligible entropy of the prefix $T_{Z^{n}}$ of the colorings of $S_{n}$. The proof of Lemma A2 is given in Appendix A.5. The proof of Lemma A3 is given in Appendix A.4 and relies on the decomposition $G_{\left[n\right]}={⨆}_{Q_{n}\in \Delta_{n}\left(Z\right)}G_{\left[n\right]}^{Q_{n}}$, where $G_{\left[n\right]}^{Q_{n}}$ is the subgraph induced by the vertices $\left(x^{n},z^{n}\right)$ such that the type of $z^{n}$ is $Q_{n}$. We show that $G_{\left[n\right]}^{Q_{n}}$ is a disjoint union of isomorphic graphs whose chromatic entropy is given by Lemma A2 and (17): $|H_{\chi }\left(G_{\left[n\right]}^{Q_{n}}\right)-n\sum_{z\in Z}Q_{n}\left(z\right)H_{\kappa }\left(G_{z}^{f}\right)|\leq n\epsilon_{n}$. Finally, uniform convergence arguments enable us draw a conclusion.

Now, let us combine these results together as follows:

(A17) $$\begin{array}{cc} R^{*}\left(g\right) & =\frac{1}{n}H_{\chi }\left(G_{\left[n\right]}\right)+o\left(1\right) \end{array}$$

(A18) $$\begin{array}{cc} \; & =\frac{1}{n}\underset{\begin{array}{c} c\;\mathrm{coloring}\;\mathrm{of}\;G_{\left[n\right]}\\ s.t.\;c\in S_{n} \end{array}}{\inf}H\left(c\left(X^{n},Z^{n}\right)\right)+o\left(1\right) \end{array}$$

(A19) $$\begin{array}{cc} \; & =\sum_{z\in Z}P_{g(Y)}\left(z\right)H_{\kappa }\left(G_{z}^{f}\right)+o\left(1\right), \end{array}$$

where (A17) comes from Theorem 1, (A18) comes from Lemma A1, and (A19) comes from Lemma A3. The proof of Theorem 3 is complete.

### Appendix A.3. Proof of Lemma A1

Let $c_{n}^{*}$ be the coloring of $G_{\left[n\right]}$ with minimal entropy. Then, we have the following:

(A20) $$\begin{array}{cc} H_{\chi }\left(G_{\left[n\right]}\right) & =\underset{c\;\mathrm{coloring}\;\mathrm{of}\;G_{\left[n\right]}}{\inf}H\left(c\left(X^{n},Z^{n}\right)\right) \end{array}$$

(A21) $$\begin{array}{cc} \; & \leq \underset{\begin{array}{c} c\;\mathrm{coloring}\;\mathrm{of}\;G_{\left[n\right]}\\ s.t.\;c\in S_{n} \end{array}}{\inf}H\left(c\left(X^{n},Z^{n}\right)\right) \end{array}$$

(A22) $$\begin{array}{cc} \; & =\underset{\begin{array}{c} c:(x^{n},z^{n})\\ \mapsto (T_{z^{n}},\tilde{c}\left(x^{n},z^{n}\right)) \end{array}}{\inf}H\left(T_{Z^{n}},\tilde{c}\left(X^{n},Z^{n}\right)\right) \end{array}$$

(A23) $$\begin{array}{cc} \; & \leq H\left(T_{Z^{n}}\right)+H\left(c_{n}^{*}\left(X^{n},Z^{n}\right)\right) \end{array}$$

(A24) $$\begin{array}{cc} \; & =H_{\chi }\left(G_{\left[n\right]}\right)+O\left(\log n\right), \end{array}$$

where (A22) comes from Definition A2; (A23) comes from the subadditivity of the entropy, $\left(x^{n},z^{n}\right)\mapsto \left(T_{z^{n}},c_{n}^{*}\left(x^{n},z^{n}\right)\right)$ is a coloring of $G_{\left[n\right]}$ that belongs to $S_{n}$, and (A24) comes from $H\left(T_{Z^{n}}\right)=O\left(\log n\right)$, as $\log|\Delta_{n}\left(Z\right)|=O\left(\log n\right)$. The desired equality comes from the bounds $H_{\chi }\left(G_{\left[n\right]}\right)$ and $H_{\chi }\left(G_{\left[n\right]}\right)+O\left(\log n\right)$ on (A21).

### Appendix A.4. Proof of Lemma A3

For all $Q_{n}\in \Delta_{n}\left(Z\right)$, let

(A25) $$\begin{array}{c} G_{\left[n\right]}^{Q_{n}}=\underset{\begin{array}{c} z^{n}\in Z^{n}\\ T_{z^{n}}=Q_{n} \end{array}}{⨆}\underset{t\leq n}{⋁}G_{z_{t}}^{f}, \end{array}$$

with the probability distribution induced by $P_{X,Z}^{n}$. This graph is formed of the connected components of $G_{\left[n\right]}$ whose corresponding $z^{n}$ has type $Q_{n}$. We need to find an equivalent for $H_{\chi }\left(G_{\left[n\right]}^{Q_{n}}\right)$. Since $G_{\left[n\right]}^{Q_{n}}$ is a disjoint union of isomorphic graphs, we can use Lemma A2 as follows:

(A26) $$\begin{array}{cc} \; & H_{\chi }\left(G_{\left[n\right]}^{Q_{n}}\right)=H_{\chi }\left(\underset{z\in Z}{⋁}{\left(G_{z}^{f}\right)}^{\vee nQ_{n}\left(z\right)}\right). \end{array}$$

On one hand,

(A27) $$\begin{array}{cc} H_{\chi }\left(\underset{z\in Z}{⋁}{\left(G_{z}^{f}\right)}^{\vee nQ_{n}\left(z\right)}\right) & \geq H_{\kappa }\left(\underset{z\in Z}{⋁}{\left(G_{z}^{f}\right)}^{\vee nQ_{n}\left(z\right)}\right) \end{array}$$

(A28) $$\begin{array}{cc} \; & =n\sum_{z\in Z}Q_{n}\left(z\right)H_{\kappa }\left(G_{z}^{f}\right), \end{array}$$

where (A27) comes from $H_{\kappa }\leq H_{\chi }$ Lemma 14 in [14], (A28) comes from (17). On the other hand,

(A29) $$\begin{array}{cc} \;\;\;H_{\chi }\left(\underset{z\in Z}{⋁}{\left(G_{z}^{f}\right)}^{\vee nQ_{n}\left(z\right)}\right) & \leq \sum_{z\in Z}Q_{n}\left(z\right)H_{\chi }\left({\left(G_{z}^{f}\right)}^{\vee n}\right) \end{array}$$

(A30) $$\begin{array}{cc} \; & =n\sum_{z\in Z}Q_{n}\left(z\right)H_{\kappa }\left(G_{z}^{f}\right)+n\epsilon_{n}, \end{array}$$

where $\epsilon_{n}≐\max_{z}\frac{1}{n}H_{\chi }\left({\left(G_{z}^{f}\right)}^{\vee n}\right)-H_{\kappa }\left(G_{z}^{f}\right)$ is a quantity that does not depend on $Q_{n}$ and satisfies $\lim_{n\to \infty }\epsilon_{n}=0$; (A29) comes from the subadditivity of $H_{\chi }$. Combining Equations (A26), (A28), and (A30) yields

(A31) $$\begin{array}{c} \left|H_{\chi }\left(G_{\left[n\right]}^{Q_{n}}\right)-n\sum_{z\in Z}Q_{n}\left(z\right)H_{\kappa }\left(G_{z}^{f}\right)\right|\leq n\epsilon_{n}. \end{array}$$

Now, we have an equivalent for $H_{\chi }\left(G_{\left[n\right]}^{Q_{n}}\right)$.

(A32) $$\begin{array}{cc} \; & \underset{\begin{array}{c} c\;\mathrm{coloring}\;\mathrm{of}\;G_{\left[n\right]}\\ s.t.\;c\in S_{n} \end{array}}{\inf}H\left(c\left(X^{n},Z^{n}\right)\right) \end{array}$$

(A33) $$\begin{array}{cc} = & \underset{\begin{array}{c} c:(x^{n},z^{n})\\ \mapsto (T_{z^{n}},\tilde{c}\left(x^{n},z^{n}\right)) \end{array}}{\inf}H\left(\tilde{c}\left(X^{n},Z^{n}\right)|T_{Z^{n}}\right)+H\left(T_{Z^{n}}\right) \end{array}$$

(A34) $$\begin{array}{cc} = & \underset{\begin{array}{c} c:(x^{n},z^{n})\\ \mapsto (T_{z^{n}},\tilde{c}\left(x^{n},z^{n}\right)) \end{array}}{\inf}\sum_{Q_{n}\in \Delta_{n}\left(Z\right)}P_{T_{Z^{n}}}\left(Q_{n}\right)H\left(\tilde{c}\left(X^{n},Z^{n}\right)|T_{Z^{n}}=Q_{n}\right)+O\left(\log n\right) \end{array}$$

(A35) $$\begin{array}{cc} = & \sum_{Q_{n}\in \Delta_{n}\left(Z\right)}P_{T_{Z^{n}}}\left(Q_{n}\right)\underset{c_{Q_{n}}\;\mathrm{coloring}\;\mathrm{of}\;G_{\left[n\right]}^{Q_{n}}}{\inf}H\left(c_{Q_{n}}\left(X^{n},Z^{n}\right)|T_{Z^{n}}=Q_{n}\right)+O\left(\log n\right) \end{array}$$

(A36) $$\begin{array}{cc} = & \sum_{Q_{n}\in \Delta_{n}\left(Z\right)}P_{T_{Z^{n}}}\left(Q_{n}\right)H_{\chi }\left(G_{\left[n\right]}^{Q_{n}}\right)+O\left(\log n\right) \end{array}$$

(A37) $$\begin{array}{cc} =\; & \sum_{Q_{n}\in \Delta_{n}\left(Z\right)}P_{T_{Z^{n}}}\left(Q_{n}\right)\left(n\sum_{z\in Z}Q_{n}\left(z\right)H_{\kappa }\left(G_{z}^{f}\right)\pm n\epsilon_{n}\right)+O\left(\log n\right) \end{array}$$

(A38) $$\begin{array}{cc} =\; & n\sum_{Q_{n}\in \Delta_{n}\left(Z\right)}2^{-nD\left(Q_{n}∥P_{g(Y)}\right)+o\left(n\right)}\left(\sum_{z\in Z}Q_{n}\left(z\right)H_{\kappa }\left(G_{z}^{f}\right)\right)\pm n\epsilon_{n}+O\left(\log n\right) \end{array}$$

(A39) $$\begin{array}{cc} =\; & n\sum_{z\in Z}P_{g(Y)}\left(z\right)H_{\kappa }\left(G_{z}^{f}\right)+o\left(n\right), \end{array}$$

where (A34) comes from $H\left(T_{Z^{n}}\right)=O\left(\log n\right)$, as $\log|\Delta_{n}\left(Z\right)|=O\left(\log n\right)$; (A35) follows from the fact that the entropy of $\tilde{c}$ can be minimized independently on each $G_{\left[n\right]}^{Q_{n}}$; (A36) follows from the definition of $G_{\left[n\right]}^{Q_{n}}$; (A37) comes from (A31); (A38) comes from Lemma 2.6 in [19] and the fact that $\epsilon_{n}$ does not depend on $Q_{n}$.

### Appendix A.5. Proof of Lemma A2

Let ${\left({\tilde{G}}_{i}\right)}_{i\leq N}$ be isomorphic probabilistic graphs and *G* such that $G={⨆}_{i}{\tilde{G}}_{i}$. Let $c_{1}^{*}:V_{1}\to C$ be the coloring of ${\tilde{G}}_{1}$ with minimal entropy, and let $c^{*}$ be the coloring of *G* defined by

(A40) $$\begin{array}{cc} c^{*}:\; & V\to C \end{array}$$

(A41) $$\begin{array}{cc} \; & vs.\mapsto c_{1}^{*}\circ \psi_{i_{v}\to 1}\left(v\right), \end{array}$$

where $i_{v}$ is the unique integer such that $v\in V_{i_{v}}$ and $\psi_{i_{v}\to 1}:V_{i_{v}}\to V_{1}$ is an isomorphism between ${\tilde{G}}_{i_{v}}$ and ${\tilde{G}}_{1}$. In other words, $c^{*}$ applies the same coloring pattern $c_{1}^{*}$ on each connected component of *G*. We have

(A42) $$H_{\chi }\left(G\right)\leq H(c^{*}\left(V\right))$$

(A43) $$\begin{array}{cc} \; & =h\left(\sum_{j\leq N}P_{i_{V}}\left(j\right)P_{c^{*}\left(V_{j}\right)}\right) \end{array}$$

(A44) $$\begin{array}{cc} \; & =h\left(\sum_{j\leq N}P_{i_{V}}\left(j\right)P_{c_{1}^{*}\left(V_{1}\right)}\right) \end{array}$$

(A45) $$\begin{array}{cc} \; & =H(c_{1}^{*}\left(V_{1}\right)) \end{array}$$

(A46) $$\begin{array}{cc} \; & =H_{\chi }\left({\tilde{G}}_{1}\right), \end{array}$$

where *h* denotes the entropy of a distribution; (A44) comes from the definition of $c^{*}$; and (A46) comes from the definition of $c_{1}^{*}$.

Now, let us prove the upper bound on $H_{\chi }\left({\tilde{G}}_{1}\right)$. Let *c* be a coloring of *G*, and let $i^{*}≐{\mathrm{argmin}}_{i}H\left(c\left(V_{i}\right)\right)$ (i.e., $i^{*}$ is the index of the connected component for which the entropy of the coloring induced by *c* is minimal). We have

(A47) $$H(c(V))=h\left(\sum_{j\leq N}P_{i_{V}}\left(j\right)P_{c(V_{j})}\right)$$

(A48) $$\begin{array}{cc} \; & \geq \sum_{j\leq N}P_{i_{V}}\left(j\right)h\left(P_{c(V_{j})}\right) \end{array}$$

(A49) $$\begin{array}{cc} \; & \geq \sum_{j\leq N}P_{i_{V}}\left(j\right)H\left(c\left(V_{i^{*}}\right)\right) \end{array}$$

(A50) $$\begin{array}{cc} \; & \geq H_{\chi }\left({\tilde{G}}_{i^{*}}\right), \end{array}$$

(A51) $$\begin{array}{cc} \; & =H_{\chi }\left({\tilde{G}}_{1}\right), \end{array}$$

where (A48) follows from the concavity of *h*; (A49) follows from the definition of $i^{*}$; (A50) comes from the fact that *c* induces a coloring of ${\tilde{G}}_{i^{*}}$; (A51) comes from the fact that ${\tilde{G}}_{1}$ and ${\tilde{G}}_{i^{*}}$ are isomorphic. Now, we can combine the bounds (A46) and (A51): for all coloring *c* of *G* we have

(A52) $$\begin{array}{c} H_{\chi }\left(G\right)\leq H_{\chi }\left({\tilde{G}}_{1}\right)\leq H\left(c\left(V\right)\right), \end{array}$$

which yields the desired equality when taking the infimum over *c*.
